# Association of Family History of Epilepsy with Earlier Age Onset of Juvenile Myoclonic Epilepsy

**Published:** 2016

**Authors:** Mohammad Reza NAJAFI, Mohammad Amin NAJAFI, Ali SAFAEI

**Affiliations:** 1 Department of Neurology, Isfahan University of Medical Sciences, Isfahan, Iran; 2 Medical student, Isfahan University of Medical Sciences, Faculty of Medicine, Isfahan, Iran; 3 Isfahan Neurosciences Research Centre (INRC), Isfahan University of Medical Sciences, Isfahan, Iran

**Keywords:** Epilepsy, Juvenile Myoclonic Epilepsy, Family history, Timeline, Age onset

## Abstract

**Objective:**

Juvenile myoclonic epilepsy (JME) is supposedly the most frequent subtype of idiopathic generalized epilepsies (IGE). The aim of this study was to determine the prevalence of JME and comparison of patients’ demographics as well as timeline of the disease between positive family history epileptic patients (PFHE) and negative family history epileptic patients (NFHE) among sample of Iranian epileptic patients.

**Materials & Methods:**

From Feb. 2006 to Oct. 2009, 1915 definite epileptic patients (873 females) referred to epilepsy clinics in Isfahan, central Iran, were surveyed and among them, 194 JME patients were diagnosed. JME was diagnosed by its specific clinical and EEG criteria. Patients were divided into two groups as PFHE and NFHE and data were compared between them.

**Results:**

JME was responsible for 10% (194 patients) of all types of epilepsies. Of JME patients, 53% were female. In terms of family history of epilepsy, 40% were positive. No significant differences was found between PFHE and NFHE groups as for gender (P>0.05). Age of epilepsy onset was significantly earlier in PFHE patients (15 vs. 22 yr, P<0.001). Occurrence of JME before 18 yr old among PFHE patients was significantly higher (OR=2.356, P=0.007).

**Conclusion:**

A family history of epilepsy might be associated with an earlier age of onset in patients with JME.

## Introduction

Millions of people are affected by epilepsy worldwide (1). Epilepsy, which involves recurrent seizures due to abnormal firing patterns of neurons within neuronal networks (2, 3), is described as a heterogeneous group of disorders caused by interactions between many genes and environmental factors (4). 

Juvenile myoclonic epilepsy (JME) is a primary generalized epilepsy syndrome classified among the idiopathic generalized epilepsies (IGEs) in accordance with the International League against Epilepsy (ILAE) classification system (5). The family history of different types of epilepsy as a possible risk factor for JME is thus, suggesting probable genetic basis of JME (6, 7). JME among siblings suggests both polygenic and autosomal recessive mode of inheritance but is of controversial subject (6, 8).

Role of family history in time line of JME still remains unclear. However, the family history of epilepsy affected the timeline of the disease (9, 10). Furthermore there are no studies available to assess the role of family history of epilepsy (FHE) in JME among Iranian patients, and particularly on the timeline of JME. 

The aim of this study was to comparison patients’ demographics and timeline of JME between positive family history epileptic patients (PFHE) and negative family history epileptic patients (NFHE).

## Materials & Methods

In this retrospective study, from Feb. 2006 to Oct. 2009, 1915 definite epileptic patients (873 females and 1042 males) referred to epilepsy clinics in Isfahan, central Iran, among them, 194 JME patients were diagnosed. The diagnosis of JME was based on strict clinical and EEG features proposed by the ILAE (11).

Electroencephalogram (EEG) pattern of myoclinic seizure shows a normal background with frequent generalized polyspike and wave discharges that may be anteriorly dominant or diffuse (By definition, polyspike and wave discharges have at least 3 spikelike components in them) (12).

The study design was approved by Ethics Committee of Isfahan University of Medical Sciences and all patients gave informed consent prior to take part in this trial. Inclusion criteria were as follows: 1) Clinical evidence of generalized seizures of myoclonic jerks with or without absence seizures; 2) No proof of neurological or intellectual impairment; 3) More than one abnormality in EEG with generalized spike-wave or polyspike-wave (SW/PSW) discharges; and 4) Normal brain imaging (CT/MRI) if performed.

Patients were excluded if encompassed the following criteria: 1) If a focal neurological or intellectual impairment were observed; 2) If there was an abnormality in imaging; 3) If myoclonic jerks was secondary to cerebral hypoxia, metabolic or degenerative diseases such as Alzheimer or Parkinson; 4) If JME developed to the generalized tonic-clonic seizure; 5) and if the data were incomplete.

We divided our patients into two groups: PFHE and NFHE, based on the consanguinity information that the patients provided in the presence of their relative(s). 

First-degree relative was identified as being the mother, father, sibling or offspring of an epileptic patient. 

Parental consanguinity was determined as second-degree relative. Prevalence of FHE among JME patients as well as information about gender, age, age of epilepsy onset (age at the first seizure) were obtained by a neurologist expert and two mentioned groups were compared. Statistical analyses were carried out using SPSS, version 20. (Chicago, IL, USA). To assess data analysis we applied independent t-test and chi-square test. P-value of less than 0.05 was considered as a statistically significant level.

## Results

Overall, 1915 epileptic patients were assessed. Totally, 194 (10%) JME patients were diagnosed, and 102 (53%) cases were female (Table 1). Seventy-seven cases (40%) of JME patients were positive family history, 58 (75%) had first-degree relatives and 19 (25%) had second degree relatives. Regarding the gender, there were no significant differences between PFH and NFH patients (P>0.05).

Age of patients was 26±14 yr (mean± s.d; Table 1), and age of JME onset was 19±15 yr. The age of epilepsy onset was significantly earlier in PFHE patients compared to NFHE patients (Table 1; 15±9 vs. 22±16 yr, respectively; mean±.s.d; P<0.001).

Occurrence of JME prior to age of 18 yr among PFHE patients was significantly greater in comparison to NFHE patients (Table 2; OR=2.356, 95%-CI: 1.260–4.408, P=0.007).

**Table 1.** Comparison between PFH Patients and NFH Patients: Patients’ Demographics and Age of Onset

	**Male**	**Female**	**Sum**	**Age**	**Age onset**
**Positive Family History (PFH)** **(40 %)**	38	40	78	21 SD=9	15 SD=9
**Negative Family History (NFH)** **(60%)**	54	62	116	28 SD=16	22 SD=16
**Sum**	92 (47.42%)	102 52.57%)	194	26 SD=14	19 SD=15
**P-value**(PFH vs. NFH)	p>0.05	p>0.05	-----	<0.001	<0.001

PFH: patients with family history of epilepsy

NFH: patients without family history of epilepsy

**Table 2:** Comparison between PFH Patients and NFH Patients: Occurrence of JME Under 18 Years Old

	**PFH**	**NFH**	**P-value**	**Odds ratio PFH/NFH**
**Under 18 occurrence**	58	64	0.007	2.356
**After 18 occurrence**	20	52	0.007	0.424

PFH: patients with family history of epilepsy

NFH: patients without family history of epilepsy

## Discussion

Surveying epilepsy genetically has considerably contributed to our understanding of the basic mechanisms in epileptogenesis in addition to its diagnosis and therapy (4, 13-15). Therefore, a detailed analysis of family history is of great value in evaluating epileptic patients. 

In this retrospective study we investigated 1915 epileptic patients. The prevalence of JME among our epileptic patients was 10% (194 patients). Various percentages for JME among all epilepsy types have been reported earlier (16-20). In large cohorts, JME prevalence was between 5-10% of all epilepsies (21). 

In this study, slight predominance of women was observed, as 53% vs. 47%. According to ILAE in 1989, distribution of JME between male and female was equal (22). However, recent reports present predominance of women in JME (23). A case series shows that approximately 60% of patients were women (24). 

Another population-based study also found that females outnumbered males with JME (25). These different percentages could be as a result of different population and different genetic basis of patients or maybe because of unknown role of sex hormones; however, further investigation is required.

Forty percent of our JME patients had FHE. In agreement our study, family history was reported in 20%-53% of JME patients (26-34). In Brasilia, history of epilepsy was in 53% JME patients (31). In Saudi Arabia, 48.7% prevalence of FHE was among JME patients (32). 

Recently, study carried out in Pakistan found 20% history of epilepsy in JME patients (29). These studies are presenting FHE in JME much more than most of other epilepsy types. These reports indicate the importance of family history in occurrence and prevalence of JME, led to significant genetic studies (30). On the other hand, JME has generalized idiopathic pattern. Previous findings strongly suggest the inheritance-underlying pattern for idiopathic epilepsies (27, 35). In addition, the association between generalized and idiopathic types of epilepsy has been mentioned in previous studies (27, 36). 

Thus, association between generalized and idiopathic types of epilepsy with genetic bases is probable reason for greater rate of PFHE among JME patients but probably this is not the only cause of higher value of FHE in JME.

Seizures can occur at any age, depending on risk factors such as sleep deprivation, structural brain abnormalities and particularly genetic factors (37, 38). Yet, the role of FH in timeline of the JME is unclear. However, previous studies evaluated the role of FH in other types of epilepsy. For instance, some studies reported earlier age of onset for idiopathic and cryptogenic epilepsies in the presence of family history (27, 35). Similarly, in a retrospective study of 420 Saudi Arabian, found earlier age of epilepsy onset among patients with family history of epilepsy in comparison to patients without a family history (9). Another study investigated the age onset of seizure in patients with focal cortical dysplasia (FCD) showed that a family history of epilepsy was associated with an earlier age at seizure onset in patients with FCD (10). Our analysis shows that the age of JME onset was significantly earlier in PFHE patients compared to NFHE patients (15 vs. 22; respectively). In addition, occurrence of JME prior to age of 18 yr (before entering the adult), was approximately 2.3 fold greater in PFHE patients in comparison with NFHE patients. Thus, despite the impact of acquired factors in the occurrence of epilepsy (4), genetic factors are strong enough to change the timeline of the disease (39).

In this study, we did not investigate the relative with JME, separately; instead, we considered all types of epilepsy as a history of JME in relatives. Further studies required to evaluate the family history of JME in JME patients.

**In conclusion, **a family history of epilepsy is associated with an earlier age of onset in patients with juvenile myoclonic epilepsy, thus suggesting that the clinical presentation in these patients is affected by genetic predisposition.
